# Dermatoglyphical impressions are different between children and adolescents with normal weight, overweight and obesity: a cross-sectional study

**DOI:** 10.12688/f1000research.19471.1

**Published:** 2019-06-25

**Authors:** Adriano Alberti, Emil Kupek, Clarissa Martinelli Comim, Carina Rossoni, Myrna Alicia Ruiz Reyes, Josiane Aparecida De Jesus, Leoberto Ricardo Grigollo, Bruna Becker da Silva, Ubirajara Duarte dos Santos, Renan Souza, Gracielle Fin, Elisabeth Baretta, Rudy José Nodari Júnior

**Affiliations:** 1 Universidade do Sul de Santa Catarina, Palhoça, Santa Catarina, 88132-270, Brazil; 2 Federal University of Santa Catarina, Florianópolis, Santa Catarina, 88040-900, Brazil; 3 Universidade do Oeste de Santa Catarina, Joaçaba, Santa Catarina, 89600-000, Brazil; 4 Universidad Autónoma de Baja California, Tijuana, Baja California, 22424, Mexico

**Keywords:** Dermatoglyphic, Obesity, Child, Adolescent

## Abstract

**Background**: Obesity is a health condition that causes a great impact on public health. The aim of this study was to determine the association between dermatoglyphic characteristics and excessive weight in children and adolescents aged 10 to 19 years in the center-west region of Santa Catarina, Brazil.

**Methods**: The sample comprised of 2,172 children and adolescents aged 10 to 19 years old of both sexes and from public and private teaching networks.

**Results**: The results suggested a predictive marker of obesity, with a greater number of lines in left hand finger two (Mesql2) and a higher frequency of the whorl pattern in participants of a healthy weight, while the overweight group had a higher frequency of the radial loop pattern and the obese group had a higher frequency of the ulnar loop pattern.

**Conclusion**: It was concluded that there may be different dermatoglyphic characteristics depending on the nutritional status of children and adolescents.

## Introduction

Obesity is a matter of constant concern for global health and is considered to be the second most common cause of preventable death ( WHO, 2014). This condition has a multifactorial origin involving genetic and environmental factors ( Martínez García *et al*., 2017) and is considered one of the major problems for public health around the world. By 2014, more than 1.9 million adults were overweight. Of these, 600 million were obese. From 1980 to 2013, the number of obese and overweight people increased by 27.5% among adults and 47.1% among children, which generated even more concern ( Ng *et al*., 2014).

The predictive value of dermatoglyphic features (DGFs) in relation to a variety of diseases has been investigated for more than five decades, from the seminal work of Cummins and Midlo in 1961 ( Bhat *et al*., 2012; Sharma & Sharma, 2012; Shetty *et al*., 2016). Dermatoglyphics is the scientific study of epidermal crescent patterns and several researchers from different fields, such as biology, anthropology, genetics, and medicine, are engaged in unraveling several unknown aspects of this field ( Raniwala *et al*., 2017). In addition, dermatoglyphics has been used as a noninvasive diagnostic tool to detect or predict different medical conditions that have a fetal origin ( Wijerathne *et al*., 2016).

The reason for the association between DGFs and health is the influence of epigenetics, which affects both ( Yohannes, 2015). The formation of the former begins in the first trimester of pregnancy, during the 6th week, is completed after the 24th week of gestation and is formed according to the development and maturation of the central nervous system ( Babler, 1991; King *et al*., 2009).

Although DGFs have no causal relationship with health ( Mittal *et al*., 2012), they may be used as a marker of health problems when there are associations that are consistent with the diseases of interest, this condition being essential for effective screening ( Gupta & Karjodkar, 2013). In the case of obesity, this is a multifactorial (polygenic and environmental) condition where epigenetic factors of obesity can influence DGF patterns; therefore, these can be used as markers of obesity throughout life ( Bhardwaj *et al*., 2015).

Our aims were to determine the association between DGF characteristics and obesity in children and adolescents aged 10 to 19 years in the center-west region of the state of Santa Catarina, in the south of Brazil and investigate whether a dermatoglyphic marker can of obesity exists.

## Methods

### Study design

A cross-sectional study of children and adolescents aged 10 to 19 years, female and male, from public and private schools in the municipality of Joaçaba, Santa Catarina, Brazil. This study was submitted to the Ethics in Research (CEP) with Human Beings from Unoesc/Hust and was approved under protocol number 449.924.

### Study participants

The data belong to the laboratory evaluation database and exercise physiology measurements of the University of West Santa Catarina (Unoesc) of Joaçaba. This data storage bank has data from 3,074 individuals investigated in the school census in the years 2013 – 2014, performed by the Institute of Educational Studies and Research “Anísio Teixeira” with the purpose of monitoring the development of these children and adolescents. The inclusion criteria for this study were students aged between 10 and 19 years enrolled in a public or private network in primary or secondary education in the municipality of Joaçaba, Santa Catarina, who participated in school censuses conducted in the years 2013 – 2014. All individuals with incomplete data for variables such as weight and height, with anomalous fingerprints due to, for example, sweat or excessive dirt on the fingers, were excluded from the sample. The final sample consisted of 2,172 students, of which 1,166 were female and 1,006 were male.

According to the National Institute of Educational Studies and Research Anísio Teixeira ( INEP, 2013), of the students enrolled in primary and secondary education in 2013 in the municipality of Joaçaba, 3,193 students were enrolled in public schools and 1,733 in private schools. In 2014, 2,842 students were enrolled in the public school system and 1,839 in the private system. Based on the number of students enrolled in their respective years, the database represents 64% of the total number of students.

### Collection of demographic characteristics

Although the students were familiar with the tests performed, the protocols of each were detailed verbally by the evaluators in order to reduce the margin of error, with the exception of the dermatoglyphic test, which is only part of this study but is easy to perform.

The body mass index (BMI) tables of the Ministry of Health of Brazil ( Ministry of Health, 2011) were used to classify BMI, dividing their percentages by age and sex, thus denominating them: low weight (< 5 ^th^ percentile), healthy weight (≥ 5 ^th^ percentile and < 85 ^th^ percentile), overweight (≥ 85 ^th^ percentile and < 97 ^th^ percentile) and obese (≥ 97 ^th^ percentile), according to the World Health Organization ( WHO, 2014).

The anthropometric evaluation of children and adolescents consisted of three phases and was carried out in the following way: first, weight was measured by a single measurement in a calibrated digital scale, with a maximum capacity of 150 kilos (kg). The scale was supported on a flat, firm and smooth surface. The participant was positioned in the center of the scale, wearing the least possible clothing, barefoot, erect, feet together, arms extended along the body and looking at the horizon ( Ministry of Health, 2011). Once their balance was stable, weight was recorded in kg.

After weight was recorded, stature was measured using a vertical mobile stem stadiometer, with a scale in centimeters (cm) and an accuracy of one millimeter (mm). The patients were positioned with their backs to the instrument, barefoot, feet together, in an upright position, looking forward, with their arms extended along the body. The mobile part of the stadiometer was placed on the top of the head at the highest point and the height reading was performed ( Ministry of Health, 2011).

The BMI was calculated using the following formula that relates weight (kg) to height (meters): BMI = Weight / Height( WHO, 1995).

### Fingerprint collection and analysis

The collection of the fingerprints occurred after the collection of the other information within the schools and was collected by the researchers. The protocol proposed by Cummins and Midlo in 1961 was chosen to analyze DGF characteristics. For the capture, processing and analysis of fingerprints, a computerized process for dermatoglyphic reading was used. The Dermatoglyphic Reader consists of an optical scanner that collects and interprets the image and constructs, in binary code, a dermatoglyphic drawing, which is processed by the reader’s specific software for the treatment and reconstruction of real and binarized images in black and white, as validated by Nodari Júnior *et al*. in 2008.

After all the images have been collected, the reader user selects them one by one and defines specific points (nucleus and deltas), tracing the Galton Line, and the software, through specific algorithms, marks the intersections of the line with the digital lines. In this way, the reader provides the number of lines on each finger, as well as the type of fingerprint pattern. The software carries out this qualitative pattern identification and quantitative determination of lines, generating a Microsoft Excel worksheet containing the processed data ( Nodari-junior *et al*., 2008).

This fingerprint analysis could also have been carried out using non-proprietary methods, such as the traditional method proposed by Cummins and Midlo using ink and paper.

### Statistical analysis

**Figure 1.**
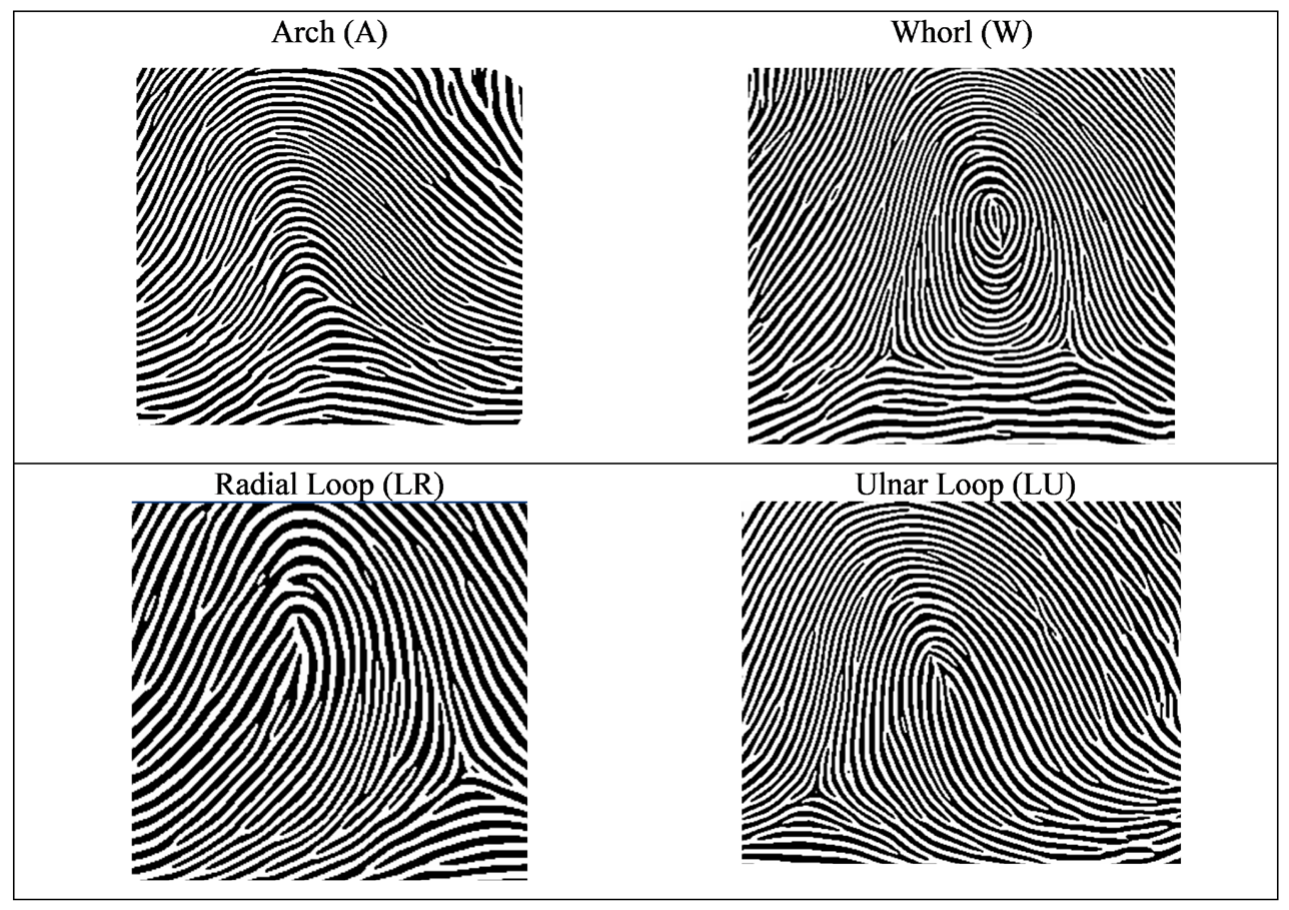
Examples of dermatoglyphic drawings. Nodari Junior and Fin authorized the reproduction of this image (taken from Nodari-Júnior & Fin, 2016), both are authors of this article.

Statistical analyzes were performed using the STATA version 12.0. Analysis of variance (ANOVA) for DGF tested the null hypothesis that there was no difference in the number of finger lines between the weight groups. The differences were considered statistically significant at p <0.05. The chi-square test was used to test whether there was a difference between weight groups in the following variables: arch, radial loop, ulnar loop and whorl fingerprint patterns ( Figure 1). The differences were considered statistically significant at p <0.05.

## Results

The final sample for this study consisted of 2,172 students, of which 1,166 were female and 1,006 were male ( Alberti, 2019). Students were aged between 10 and 19 years and enrolled in a public or private primary or secondary education in the municipality of Joaçaba, Santa Catarina-Brazil.

As for the quantitative fingerprint variables (the number of lines of each finger, the total number of lines on the fingers of the right hand, the total number of lines on the fingers of the left hand and the total number of lines on the fingers of both hands), it was observed that, on average, individuals who were of a healthy weight had a greater number of lines in relation to overweight and obese individuals in MET2 (left-hand finger two). ( Table 1).

**Table 1.** Number of finger lines by weight.

Number of lines on fingers	All (n=2162): mean (SD)	Weight status			
		Healthy weight (n=1862): mean (SD)	OW (n=204): mean (SD)	Obese (n=96): mean (SD)	p-value *
Mesql1	12.86 (5.47)	12.87 (5.47)	12.68 (5.23)	13.10 (5.88)	0.8089
Mesql2	8.68 (5.64)	8.83(5.63)	7.63(5.25)	7.98(6.34)	*0.0068
Mesql3	9.86(5.67)	9.92(5.70)	9.48(5.17)	9.48(6.20)	0.4520
Mesql4	12.37(5.61)	12.39(5.56)	12.32(5.64)	12.12(6.45)	0.8968
Mesql5	10.87(5.03)	10.87(5.02)	10.75(5.06)	11.25(5.30)	0.7150
Sqtle	54.64(21.63)	54.88(21.43)	52.85(21.72)	53.94(25.22)	0.4217
Mdsql1	14.71(5.45)	14.61(5.40)	15.41(5.54)	15.08(6.25)	0.1109
Mdsql2	9.13(5.70)	9.22(5.74)	8.47(5.27)	8.81(5.92)	0.1737
Mdsql3	10.19(5.03)	10.20(5.02)	10.39(4.85)	9.63(5.61)	0.4619
Mdsql4	12.54(5.44)	12.64(5.42)	12(5.32)	11.77(6.05)	0.1018
Mdsql5	10.97(5.08)	10.95(5.06)	11.10(5.08)	11.13(5.65)	0.8861
Sqtld	57.55(20.59)	57.63(20.35)	57.37(21.01)	56.42(24.23)	0.8468
Sqtl	112.19(41.04)	112.51(40.57)	110.23(41.75)	110.35(48.30)	0.6795

Note: *oneway ANOVA.

OW, overweight; Mdsql, number of lines on right hand fingers; Mesql, number of lines on left hand fingers; Sqtle: sum of the number of lines on the left hand; Sqtld, sum of the number of lines on the right hand; Sqtl, sum of the number of lines on both hands.

The results obtained may suggest the presence of predictive markers for BMI in the researched population. For left-hand finger two (MET2), participants of a healthy weight presented with a higher frequency the whorl pattern, while overweight and obese participants had a higher frequency of the ulnar loop pattern; for left-hand finger three (MET3), participants of a healthy weight presented a higher frequency of the radial loop pattern, while overweight and obese participants had a higher frequency of the ulnar loop pattern; for left-hand finger four (MET4), participants of a healthy weight presented a higher frequency of the whorl pattern, while the overweight group presented a higher frequency of the radial loop and the ulnar loop pattern; for left-hand finger five (MET5), participants of a healthy weight presented a higher frequency of the arch pattern, while the overweight group presented a higher frequency of the radial loop pattern and the obese group had a higher frequency of the ulnar loop pattern; for finger one of the right hand (MDT1), participants of a healthy weight presented a higher frequency of the arch pattern, the overweight group had a higher frequency of the radial loop pattern, and the obese group had a higher frequency of the ulnar loop pattern; for right-hand finger three (MDT3), participants of a healthy weight had a higher frequency of the whorl pattern, while overweight and obese groups had a higher frequency of the ulnar loop pattern; for right-hand finger four (MDT4), participants of a healthy weight had the highest frequency of the whorl pattern, while the overweight group presented a higher frequency of the radial loop pattern and the obese group presented a higher frequency of the figure ulnar loop; for right-hand finger five (MDT5), participants of a healthy weight presented a high frequency of the whorl pattern, while the overweight group presented a high frequency of the radial loop pattern and the obese group presented a high frequency of the ulnar loop pattern. ( Table 2).

**Table 2.** Finger line patterns by weight status.

Hand / Finger	Type of dermatoglyphic figures	Weight status			p-value *
		Healthy weight (%)	OW (%)	Obese (%)	
Left hand, finger 1 MET1	Arch	86.58	9.74	3.67	0.205
	Radial Loop	80.00	20.00	0.00	
	Ulnar Loop	81.81	12.73	5.45	
	Whorl	86.08	8.35	5.57	
Left hand, finger 2 MET2	Arch	86.31	9.28	4.41	*0.046
	Radial Loop	87.19	8.37	4.43	
	Ulnar Loop	79.63	12.04	8.33	
	Whorl	87.32	9.44	3.24	
Left hand, finger 3 MET3	Arch	87.06	8.80	4.14	*0.008
	Radial Loop	94.29	2.86	2.86	
	Ulnar Loop	78.57	13.27	8.16	
	Whorl	84.57	11.43	4.00	
Left hand, finger 4 MET4	Arch	85.65	9.57	4.78	*0.002
	Radial Loop	81.25	18.75	0.00	
	Ulnar Loop	73.17	17.07	9.76	
	Whorl	88.41	8.06	3.53	
Left hand, finger 5 MET5	Arch	86.56	9.21	4.23	*0.000
	Radial Loop	50.00	50.00	0.00	
	Ulnar Loop	72.41	13.79	13.79	
	Whorl	88.12	7.92	3.96	
Right hand, finger 1 MDT1	Arch	87.37	8.30	4.33	*0.005
	Radial Loop	66.67	33.33	0.00	
	Ulnar Loop	74.19	16.13	9.68	
	Whorl	85.62	10.09	4.29	
Right hand, finger 2 MDT2	Arch	85.71	9.52	4.76	0.391
	Radial Loop	89.53	7.56	2.91	
	Ulnar Loop	83.04	10.71	6.25	
	Whorl	85.96	9.83	4.21	
Right hand, finger 3 MDT3	Arch	86.21	9.51	4.28	*0.032
	Radial Loop	83.33	11.11	5.56	
	Ulnar Loop	77.59	12.07	10.34	
	Whorl	89.02	7.93	3.05	
Right hand, finger 4 MDT4	Arch	85.37	9.59	5.04	*0.000
	Radial Loop	78.95	21.05	0.00	
	Ulnar Loop	76.00	8.00	16.00	
	Whorl	88.15	8.77	3.08	
Right hand, finger 5 MDT5	Arch	86.37	9.27	4.37	*0.002
	Radial Loop	69.23	30.77	0.00	
	Ulnar Loop	76.00	16.00	8.00	
	Whorl	88.46	6.73	4.81	

Note: *chi-square test. OW, overweight; MET, left hand; MDT, right hand.

## Discussion

This study suggests that a higher number of the total number of lines in Mesql2 may be a predictive marker of obesity and that a higher frequency of the whorl pattern may be found in people of a healthy weight, a higher frequency of the radial loop pattern may be found in overweight people and a higher frequency of the ulnar loop pattern may be found in obese people.

An increase in weight in children and adolescents can occur rapidly, causing, for example, low levels of cardiorespiratory and musculoskeletal aptitude and impairing the quality of life of these individuals ( García-Hermoso *et al*., 2018). Several studies have begun to recognize epigenetic factors in obesity and, despite a relatively high heritability of non-syndromic common obesity (40–70%), the search for genetic variants that contribute to susceptibility has been a challenging task. Genome-wide association studies have dramatically changed the pace of detection of common variants involved in genetic susceptibility. By the year 2011, more than 40 genetic variants were associated with obesity. However, since these variants do not fully explain the heritability of obesity, other forms of variation, such as epigenetic markers, should be considered ( Herrera *et al*., 2011).

Every organism is unique and has epigenetic traits that are inherited and generated in the womb. Studies have been conducted that are aimed at highlighting the influence of the gestation period and fetal environment for the development of diseases and conditions over a lifetime, such as obesity ( Martínez García *et al*., 2017). The fetal development phase begins at the 9th week of gestation and goes through to the baby’s birth, the human gestation lasting on average 38 weeks ( Dipietro, 2008). There are studies that reinforce that epigenetic influences have a strong association with the development of obesity ( Ornellas *et al*., 2017).

Dermatoglyphics has its fundamental basis in this premise, being an epigenetic marker related to the period of fetal development ( Yohannes, 2015). In addition to the fact that the fingerprints are intrinsically related to the central nervous system and can therefore reflect motor capacities inherited genetically and epigenetically for conditions that may have a marker expressed during this period of fetal development, fingerprint evaluation is a simple and practical method ( King *et al*., 2009).

In one sample of 370 obese children, in a study to identify dermatoglyphic patterns in obese individuals and to discover the association between standard dermatoglyphics and obesity, a high frequency of the arch pattern was observed in the right thumb ( Bhardwaj *et al*., 2015).

In another study, the authors ( Oladipo *et al*., 2010) sought to determine the dermatoglyphic characteristics of obese Nigerian patients by comparing a group of 50 obese individuals (25 men and 25 women) with a group of 50 normal weight subjects (25 men and 25 women). The arch pattern was observed in the first digits of the right hand in 54.5% of obese men and 42.33% of obese women, whereas individuals with normal weight presented the figure more frequently.

In the city of São Paulo, Brazil, a survey ( Pasetti *et al*., 2012) with 30 obese Brazilian women with a mean age of 46.1 ± 07.87 years, all with a BMI equal to or greater than 30, observed that participants presented a high frequency of the arch pattern, low frequencies of the ulnar loop pattern and a high frequency of the whorl pattern. These results corroborate the findings of several other authors ( Bhardwaj *et al*., 2015; Oladipo *et al*., 2010) who also presented a predominance of the arch pattern in the obese group.

The present study utilized a sample of 2,172 individuals and the computerized method developed by Nodari Júnior, Heberle, Ferreira-Emygdio and Irany-Knackfuss in 2008, providing greater precision in the dermatoglyphic analysis. This method allows optimization of the analysis and greater reliability in the counting and marking of lines and designs. It allowed differentiation of the ulnar loop and radial loop patterns, which other studies in dermatoglyphics and obesity have not done.

The results showed the presence of different dermatoglyphic characteristics for different nutritional statuses of children and adolescents, indicating a higher number of the total number of lines in Mesql2 and a higher frequency of the whorl pattern may be found in people of a healthy weight, a higher frequency of the radial loop pattern may be found in overweight people and a higher frequency of the ulnar loop pattern may be found in obese people.

This data may contribute to this field of research and allow better and more adequate referrals possible for people that have a predictive marker of fetal origin of obesity.

As a limitation, because it was a cross-sectional study, it was not possible to associate the results with important factors, such as prenatal and family history, and it is recommended in future studies that a cohort-type follow-up should be performed to verify a possible association between these factors and the level of physical activity, along with fingerprints.

## Data availability

### Underlying data

Open Science Framework: Data file new 5_Dermatoglyphical impressions are different between children and adolescents with normal weight, overweight and obesity https://doi.org/10.17605/OSF.IO/AFN62 ( Alberti, 2019)

This project contains the following underlying data:

- Data file new 5_Dermatoglyphical impressions are different between children and adolescents with normal weight, overweight and obesity.xlsx (demographic information, the number of finger lines and fingerprint pattern types for each participant)

Data are available under the terms of the Creative Commons Zero “No rights reserved” data waiver (CC0 1.0 Public domain dedication).
