# Supplementary material for: Dermatoglyphical impressions are different between children and adolescents with normal weight, overweight and obesity: a cross-sectional study
Source: F1000Res. 2019 Jun 25;8:964. [Version 1] doi: 10.12688/f1000research.19471.1 (PMC7331104; doi:10.12688/f1000research.19471.1)
Supplement: Peer review history

# Peer review: Reviewer response for version 1

**DOI:** 10.5256/f1000research.21350.r62596

**Published:** 2020-07-01

**Authors:** Jannu Chiranjeevi

**Affiliations:** 1 Vaagdevi College of Physiotherapy, Warangal, Telangana, India

Firstly I congratulate the authors for doing great work on the title "Dermatoglyphical impressions are different between children and adolescents with normal weight, overweight and obesity: a cross-sectional survey."

**Introduction:**

- The introduction was very clear and the authors have clearly described obesity and dermatoglyphic features in relation to a variety of diseases. The aim of the study was well determined about obesity in children and adolescents aged 10 to 19 years in the centre west region of Santa Catarina.

**Methods:**

- Under this section, the study design and study participants were well documented. But I feel that the census was made only between 2013 - 2014. It could be more productive if they have done in at least 5 years census. The collection of demographic characteristics and fingerprint collection and analysis within the schools was done properly by the authors. 
- Statistical analysis using ANOVA was well apt to the study and the results were well documented under the results section. The table and figures are giving proper information about the study.

**Discussion:**

- The whole study was well summarised under this section. The limitations of the study were well documented.

Overall it's a very good study which is most beneficial.

Is the work clearly and accurately presented and does it cite the current literature?

Yes

If applicable, is the statistical analysis and its interpretation appropriate?

Yes

Are all the source data underlying the results available to ensure full reproducibility?

Yes

Is the study design appropriate and is the work technically sound?

Yes

Are the conclusions drawn adequately supported by the results?

Yes

Are sufficient details of methods and analysis provided to allow replication by others?

Yes

Reviewer Expertise:

Physical therapy, Physiotherapy, Neurosciences, Rehabilitation, ergonomics, stroke, cerebral palsy, spinal cord injuries, diabetes, diabetic neuropathy

I confirm that I have read this submission and believe that I have an appropriate level of expertise to confirm that it is of an acceptable scientific standard.

# Peer review: Reviewer response for version 1

**DOI:** 10.5256/f1000research.21350.r64349

**Published:** 2020-06-24

**Authors:** Ali Azhar Dawasaz

**Affiliations:** 1 Department of Diagnostic Sciences and Oral Biology, College of Dentistry, King Khalid University, Abha, Saudi Arabia

The authors have tried to address the relationship between dermatoglyphics and nutritional status (normal, overweight, and obese) in participants aged 10 to 19 years from the center-west region of Santa Catarina, Brazil using BMI. This is a cross-sectional study on a large sample population and addresses an important health parameter like obesity that is not only local but a global phenomenon. Dermatoglyphics is a relatively easier screening tool that is gaining importance in identifying health-related risks. This article is an important addition to the current literature.

 Some concerns however remain and need some additional information from the authors.

1. I would like to ask authors to discuss the importance of the use of BMI when there are other more reliable tools to screen individuals’ health statuses. BMI is considered a macro tool and can be used to calculate a baseline value for an ethnically identified population. However, BMI carries inherent flaws when incorporated in a correlation study where a mixed population is under investigation.
2. Introduction section, line 6 says: By 2014, more than 1.9 million adults were overweight. Of these, 600 million were obese. Please correct the discrepancy in figures and provide a valid reference.
3. Page 4, under BMI calculation, the formula needs to be corrected.
  BMI=Weight in kg/ (Height in mtr) ^2^
4. Please provide name and company details of the commercially available dermatoglyphic reader used in this study.
5. The weight of participants was noted on a digital scale nonetheless, considering variations caused due to intake of food or water prior to measurement would give varying results and could impact the reliability of data observed. It is requested that the authors kindly add an additional statement regarding the above concern whether taken into consideration or not.
6. In general, the objectives and study design are acceptable; however, the conclusions drawn from the study allowing prediction of obesity using DGF remains unclear. Authors are requested to carry out additional statistical tests (Regression/correlation) in order to have a compelling reason to conclude the same.

Is the work clearly and accurately presented and does it cite the current literature?

Yes

If applicable, is the statistical analysis and its interpretation appropriate?

Partly

Are all the source data underlying the results available to ensure full reproducibility?

Partly

Is the study design appropriate and is the work technically sound?

Yes

Are the conclusions drawn adequately supported by the results?

Partly

Are sufficient details of methods and analysis provided to allow replication by others?

Yes

Reviewer Expertise:

Dermatoglyphics, Radiation hazards, dentistry,

I confirm that I have read this submission and believe that I have an appropriate level of expertise to confirm that it is of an acceptable scientific standard, however I have significant reservations, as outlined above.

## References

[1] : Socioeconomic modifiers of genetic and environmental influences on body mass index in adult twins. *Health Psychol*.2016;35(2) : 10.1037/hea0000255 157-66 10.1037/hea0000255 26348497PMC4913513

[2] : Dermatoglyphics from all Chinese ethnic groups reveal geographic patterning. *PLoS One*.2010;5(1) : 10.1371/journal.pone.0008783 e8783 10.1371/journal.pone.0008783 20098698PMC2808343

[3] : Can Dermatoglyphics Be Used as a Marker for Predicting Future Malocclusions?. *Electron Physician*.2016;8(2) : 10.19082/1927 1927-32 10.19082/1927 27054000PMC4821306

[4] : Three-dimensional palatal anatomic characteristics' correlation with dermatoglyphic heterogeneity in Angle malocclusions. *Angle Orthod*.89(4) : 10.2319/091718-675.1 643-650 10.2319/091718-675.1 30840497PMC8117199

# Peer review: Reviewer response for version 1

**DOI:** 10.5256/f1000research.21350.r62253

**Published:** 2020-04-29

**Authors:** Prathap Lavanya, Prathap Suganthirababu

**Affiliations:** 1 Department of Anatomy, Saveetha Dental College and Hospitals, Saveetha Institute of Medical and Technical Sciences, Chennai, Tamil Nadu, India; 2 Neurological Physiotherapy, Saveetha Institute of Medical and Technical Sciences, Chennai, Tamil Nadu, India

The author has attempted to associate the genetic and epigenetic influence of obesity and overweight which is represented in dermatoglyphic patterns. The author’s interest in predicting the epigenetic influence of obesity through a non-invasive, cost – effective way of analysis is highly appreciable. The author used dermatoglyphic scanner instead of traditional method for data collection improves the accuracy. The methodology is clear and reproducible. He used appropriate statistical tools and the results conclude significant difference between the obese, overweight and healthy population.

 Few corrections to be made –

1. Introduction section – last paragraph –last sentence - kindly check the sentence formation.
2. Under Methodology - Collection of demographic details - avoid using ‘patient’ can alternate it with ‘participants’.
3. Under Results section – Paragraph 1- The description is repeated, can avoid repetition.
4. Under results section – Paragraph – 2 - the codes used for digits are varying. Can use any one form of representation either Mesql2 or MET2.
5. Under the results section in the description, the digits are represented as MET / But in the reference table, it is represented as Mesql. Kindly fix with one form of representation and use the same in both description and data table.

 

  The article is recommended for publication after the above said corrections.

Is the work clearly and accurately presented and does it cite the current literature?

Yes

If applicable, is the statistical analysis and its interpretation appropriate?

Yes

Are all the source data underlying the results available to ensure full reproducibility?

Yes

Is the study design appropriate and is the work technically sound?

Yes

Are the conclusions drawn adequately supported by the results?

Yes

Are sufficient details of methods and analysis provided to allow replication by others?

Yes

Reviewer Expertise:

Dermatoglyphics, breast cancer, epigenetics, Human anatomy,

We confirm that we have read this submission and believe that we have an appropriate level of expertise to confirm that it is of an acceptable scientific standard.

# Peer review: Reviewer response for version 1

**DOI:** 10.5256/f1000research.21350.r51076

**Published:** 2019-08-27

**Authors:** Tarimobo Otobo

**Affiliations:** 1 Department of Diagnostic Imaging, The Hospital for Sick Children, University of Toronto, Toronto, ON, Canada; 2 Institute of Medical Sciences, University of Toronto, Toronto, ON, Canada

The authors attempted to study and report the finger dermatoglyphic characteristics of normal, overweight and obese children. The choice of cross-sectional study design for the purpose of reporting the palmer dermatoglyphics characteristics in the sample population is commendable as it provided the baseline distribution of arch, whorl ulna, and radial loops in the study population. The analysis of the distribution and comparison of the finger dermatoglyphic characteristics between the normal, overweight and obese subjects was exceptional.

 I am not sure if some of the conclusions were intended, e.g. suggesting that a particular fingerprint pattern is predictive of an outcome (in this case either of obese, overweight and normal weight) was overreaching. More so, since the study design and statistical analysis, did not provide sufficient evidence for such a conclusion.

 The correct reference of the manual ink pad dermatoglyphic collect method is by Antonok *et al.* ^1^

 It was challenging to comprehend most of the paragraphs, because of challenging lexical semantics. A detail grammatical edit is recommended.

 In general, the objective and study design of the article good; however, a major revision is required before final publication release.

Is the work clearly and accurately presented and does it cite the current literature?

Partly

If applicable, is the statistical analysis and its interpretation appropriate?

Partly

Are all the source data underlying the results available to ensure full reproducibility?

Yes

Is the study design appropriate and is the work technically sound?

No

Are the conclusions drawn adequately supported by the results?

No

Are sufficient details of methods and analysis provided to allow replication by others?

Partly

Reviewer Expertise:

Development and Validation of Outcome measurement tools, Physical anthropometry, and big data computational analytics.

I confirm that I have read this submission and believe that I have an appropriate level of expertise to state that I do not consider it to be of an acceptable scientific standard, for reasons outlined above.

## References

[1] To the method of receiving of human palmer prints. *Voprosy anthropol.*.1975;50:217-221
